# A literature review and case report of hand, foot and mouth disease in an immunocompetent adult

**DOI:** 10.1186/s13104-016-1973-y

**Published:** 2016-03-15

**Authors:** Carlos Omaña-Cepeda, Andrea Martínez-Valverde, María del Mar Sabater- Recolons, Enric Jané-Salas, Antonio Marí-Roig, José López-López

**Affiliations:** School of Dentistry, University of Los Andes, Mérida, Venezuela; Department of Odontostomatology, School of Dentistry, University of Barcelona, L’Hospitalet de Llobregat, Barcelona, Spain; Oral Health and Masticatory System Group (Bellvitge Biomedical Research Institute) IDIBELL, University of Barcelona, L’Hospitalet de Llobregat, 08907 Barcelona, Spain; Department of Oral and Maxillofacial Surgery, University Hospital Bellvitge (HUB), c/Feixa Llarga, Hospitalet de Llobregat, 08907 Barcelona, Spain; Dental Hospital Barcelona University, Universitary Campus of Bellvitge, C/Feixa LLarga S/N, L’Hospitalet de Llobregat, 08907 Barcelona, Spain

**Keywords:** HFMD, Immunocompetent, Adult, Dentistry, Oral health, Case report

## Abstract

**Background:**

To report an uncommon case of hand, foot and mouth disease, (HFMD) in an immunocompetent adult; a highly infectious disease, characterized by the appearance of vesicles on the mouth, hands and feet, associated with coxsackieviruses and enteroviruses; including a literature review.

**Case report:**

A 23 year Caucasian male with no medical or surgical history, no allergies, was not taking any medication and smoked ten cigarettes a day, suffering from discomfort in the oral cavity; itching, burning and pain when swallowing associated with small erythematous lesions located on the hard palate, and small ulcers in tonsillar pillars and right buccal mucosa. Mild fever of 37.8 °C and general malaise. The patient reported he had had contact with a child diagnosed with HFMD. From his background and symptoms, the patient was diagnosed with HFMD. Following symptomatic treatment, the symptoms remitted in 7 days.

**Methods:**

A literature review in MEDLINE (PubMed). The inclusion criteria were for studies on humans over the last 5 years, using the keywords HFMD.

**Results:**

We found 925 articles, which were subsequently reduced to 52 documents after applying the inclusion criteria. Maculopapular lesions were found on hands and feet.

**Conclusions:**

Dentists may have a key role diagnosing the disease. A surveillance system to predict future outbreaks, encourage early diagnosis, put appropriate public health measures in place and research vaccine development is vitally important in order to control the disease.

## Background

In 1958, Robinson et al. first described the outbreak of a highly infectious disease in Toronto in 1957, characterized by the appearance of vesicles on the mouth, hands and feet [1]. It was isolated coxsackievirus (CV) A16. Hand foot and mouth disease (HFMD) rarely appears as an epidemic infectious disease. However it is the most common infectious disease in China, with an incidence rate of around 500,000–1,000,000 cases per year [2]. It is associated with climate changes, usually occurring in spring and summer [3]. It occurs most often in children between 0 and 5 years old [4, 5] and immunocompromised adults [6], due to their high sensitivity to the enterovirus 71 (EV71) and CVA16 [7]. However, it can also occur in immunocompetent adults [5, 6]. Some of the major causative agents are EV71, CVA16 [8], and it was recently described, CVA6 and CVA10 [9–11].

The main routes of transmission are person-to-person (through oral-pharyngeal secretions or by direct vesicle contact), via contaminated water (fecal-oral route) [12], and via contaminated objects.

The incubation period is short, ranging from 2 to 7 days. It shows non-specific symptoms, but there may be mild fever and catarrhal manifestations. The initial viral implantation is in the oral cavity and ileum, spreading to the regional lymph nodes within 24 h. Viremia occurs after 72 h, followed by secondary infection and viral seeding in areas such as the oral mucosa, hands and feet. On the seventh day, there is an increase in antibody levels and the disease begins to disappear [13].

Oral lesions are the first clinical signs of the disease, and are sometimes the only sign, because they appear even before the lesions on the extremities [12]. Manifestations of the disease on the skin consist of multiple lesions on the hands and feet, and occur concurrently or shortly after the oral lesions. Systemic features are summarized in the Table 1 [13, 14].

**Table 1:** Systemic manifestations oh HFMD [14, 15]

Systemic features of HFMD	Systemic features in severe occurrences of HFMD
Anorexia, fever, low pollution, sore throat, runny nose, abdominal pain, and sometimes myalgia, lymphadenopathy, diarrhea, nausea and vomiting	Skin rashes, fever ≥38 °C, neurological symptoms, respiratory symptoms such as tachypnea or bradypnea, pulmonary edema, cardiovascular symptoms such as tachycardia and hypertension, bleeding, pulmonary consolidation, hyperglycemia, elevated leukocyte and high levels of lactic acid

A study in Japan suggests the possibility that the HFMD could also cause opsoclonus-myoclonus (jerky eye movements in all directions) as a possible viral or autoimmune response [15], other studies reports cases of retinopathy and vision loss in this entity [16].

The diagnosis is by observing the clinical signs of the disease, such as fever and the characteristic lesions on the hands, feet and mouth. Confirmation of diagnosis is carried out by isolating the virus responsible for the disease, or by identifying virus-neutralizing antibodies in patient serum [5].

There are varieties of studies investigating possible treatments for HFMD. One such study considers using intravenous immunoglobulin (IVIG) as therapy against HFMD [17, 18]. However, because the disease is self-limiting in nature and due to the lack of a virus-specific therapy, the present treatment is symptomatic. Non-specific rinses with anesthetic substances can be employed to relieve oral discomfort. In addition, ensure that the patient gets plenty of fluids and avoids spicy and acidic foods, and foods that require a lot of chewing.

In most cases, the prognosis is good moving toward spontaneous healing within 7–10 days without sequelae, scabs or scars. However, there have been reports of onychomadesis associated with the disease [19–21], in addition to neurological disorders such as meningitis (EV-4) [22], Guillain–Barre syndrome, meningoencephalitis, as well as paralytic polio, myoclonus and somnolence, mainly caused by the sub genotype C4aEV71 [23], in children under 5 years (the main risk group in the population) [24]. It has been shown that most of these patients have low levels of vitamin A, associated with reduced immunity, and they are therefore more susceptible to a more severe manifestation of the disease [25]. Note also that delays in access to health services lead to an increased likelihood of a more severe form of the disease [4, 26, 27].

The aim of this article is to present a case of HFMD in an immunocompetent adult, alongside a literature review. The article also aims to identify the etiologic agents and publicize transmissible factors in order to correctly diagnose patients, and thus to establish an effective treatment plan both individually and collectively.

## Case report

**Fig. 1:**
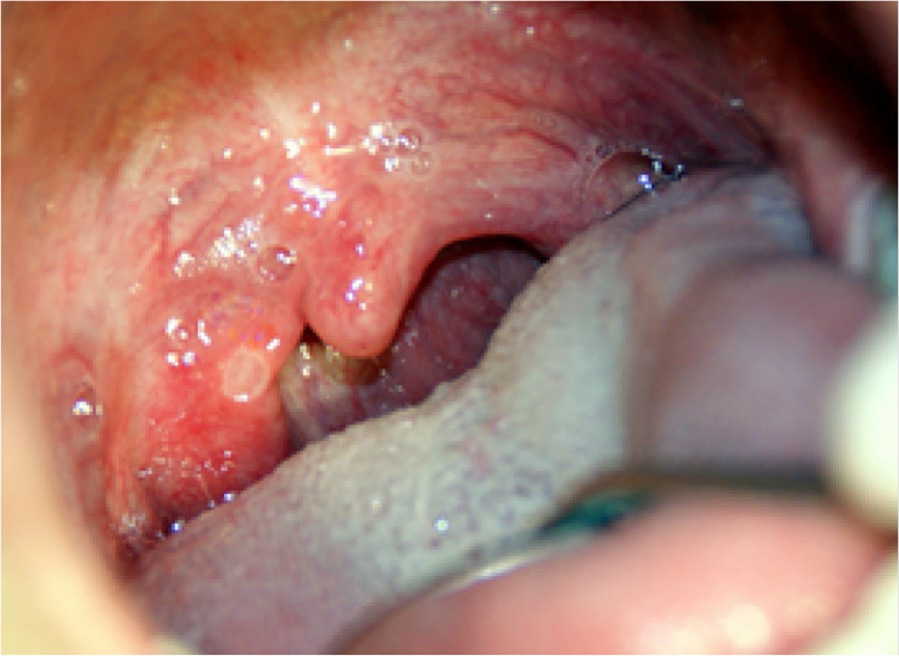
Lesions on the hard palate, soft and tonsillar pillars

**Fig. 2:**
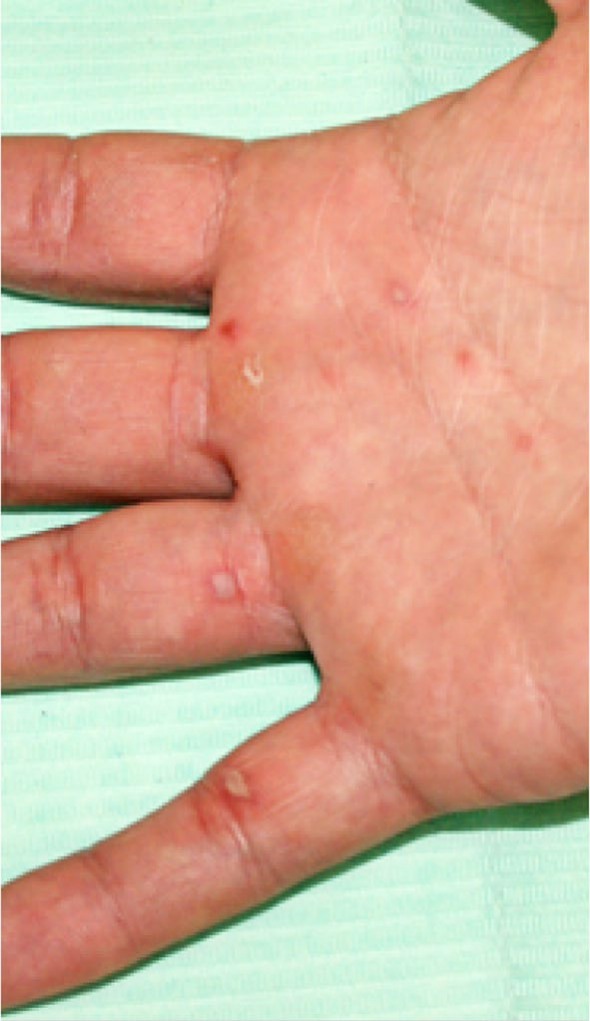
Lesions on the palm and fingers

**Fig. 3:**
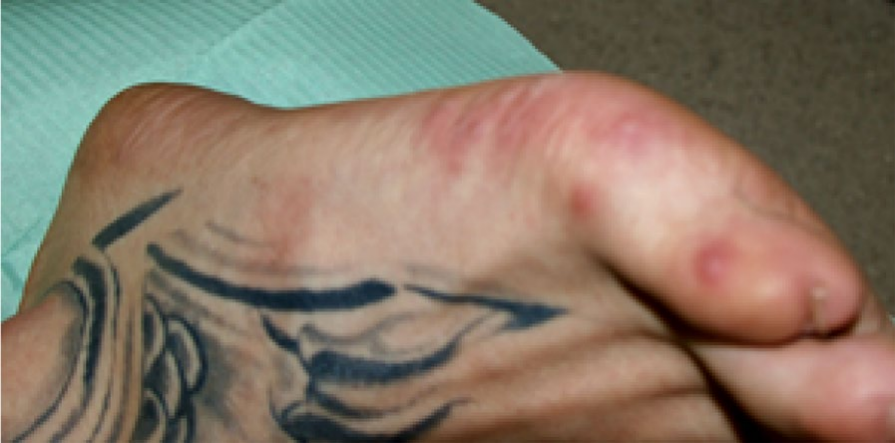
Lesions on the foot

In May 2014, coinciding with the springtime, a 23 year Caucasian male went to the Odontology Hospital of the University of Barcelona. He had no familiar, medical or surgical history, no allergies, not taking any medication and smoked ten cigarettes a day. He was suffering from discomfort in the oral cavity: itching, burning and pain when swallowing associated with small erythematous lesions located on the hard palate, and small ulcers in tonsillar pillars and right buccal mucosa. The patient had a mild fever of 37.8 °C and general malaise. The examination also found maculopapular lesions on the hands and feet, some of which were in the form of blisters (Figs. 1, 2 and 3). Upon questioning the patient, he stated that a few days earlier (7–10 days), he had been in contact with a 2-year-old girl who was diagnosed with HFMD, and that other members of the child’s family had suffered the same symptoms.

From his background and symptoms, the patient was diagnosed with HFMD and symptomatic treatment was begun with analgesics (650 mg paracetamol three times a day), hygiene, and a diet that would not irritate the symptoms further. The patient controlled the symptoms for 7 days until the disease subsided. An analytical control was requested in which no evidence of any significant alteration was shown. We conducted a follow-up visit after 15 days and did not find any residual lesions.

## Methods and results

A literature review was performed via an automated search for information on the database: MEDLINE (PubMed) to identify and summarize all relevant publications on HFMD. The search strategy was based on the terms HFMD. The inclusion criteria was for studies on humans, over the last 5 years that identify the etiologic agents, disclose communicable factors, correctly diagnose the patients, and establish an effective treatment plan both individually and collectively. We assessed the eligibility of articles from the titles and abstracts, and extracted the information related to our target subject. We also included an article by Delgado et al. [13], as well as two older articles for their importance to the history of the disease: Robinson et al. [1], and Alsop et al. [28].

**Fig. 4:**
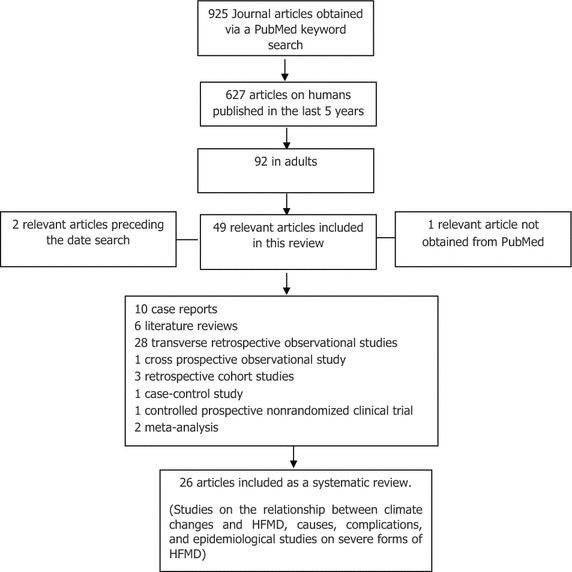
Diagram of article selection

We found 925 publications in PubMed with the keywords listed, of which 627 were based on humans and published in the last 5 years, which was reduced to 92, when we limit the search to studies in adults. We selected 49 papers that met the objectives of our research and the inclusion criteria. We included 26 of the 52 total papers as a systematic review, based in the number of cases presented and type of studies, and addressing the issues developed: the relationship between climate changes and HFMD, causes, complications and epidemiological studies on severe forms of HFMD, the remaining papers was included for the discussion and development of theme (Fig. 4).

## Discussion

HFMD is a syndrome caused by intestinal viruses from the Picornaviridae family, and is mainly characterized by the appearance of vesicular lesions on the mouth, hands and feet. It is more common in children under 10 and in vulnerable adults. Nonetheless, it can also occur in young immunocompetent adults [6, 9–11, 15], as in the case reported.

For this review, we will build on the most relevant items and features that in relation with the HFMD are described in the selected papers.

### Climate

Recent studies suggest a strong association between HFMD and climate changes, as the incidence of the disease increases in the springtime, when our case occurred. However, the exact reason for this association has not been studied yet. Different studies show contradictions in temperatures, while Wang et al. [4] provides the greatest incidence range between 21.1 and 26.6 °C. A study by Hii et al. [29] cites data above 32 °C and in periods where the temperature difference between the minimum and the maximum is greater than 7 °C. Neither is rainfall a clear risk factor, since according to Wang et al. [4] there are more cases with high rainfall, while for others [29] there is a low incidence rate 0.5 % for rainfall above 75 mm (Table 2). The biological relationship between climate indicators and EV activity is quite complicated and different reports on the subject do not reveal the same results, so it deserves to be investigated in future studies to get better information on this relationship [2, 29].

**Table 2:** Studies on the relationship between climate changes and HFMD

Study	Results	Conclusions
HanWang et al. Beijing, China 2011 [4]	Spring OR = 1.4–1.6 Other seasons OR ≤1.2 Increased risk of transmission: Temperature 21.1–26.6 °C High relative humidity Low wind speed High rainfall High population density Schools open	Strong relationship between climatic factors and the transmission of HFMD
Hii et al. Umeå, Sweden 2011 [29]	With each degree Celsius that the maximum temperature rises above 32 °C, the risk of disease incidence increases by 36 % Rainfall below 75 mm increases risk by 0.3 %. Above 75 mm, risk fell by 0.5 % Temperature differences of more than 7 °C between the minimum and maximum temperature increase the incidence rate by 41 %	The results suggest a strong association between HFMD and climate changes
Park et al. South Korea, 2010 [12]	Having a non-water closet toilet, changes in water quality, and contact with HFMD patients were associated with risk of HFMD (OR = 3.3, 2.8, 6.9, and 5.0, respectively) Visiting a hospital, changes in water quality, presence of a skin wound, eating out, and going shopping were significantly associated with the risk of HFMD (OR = 9.0, 37.0, 11.0, 12.0, 37.0, and 5.0, respectively)	The results suggest that seasonal variations, geographic localization, person to-person contact and contaminated water could be the principal modes of transmission of HFMD

*HFMD* hand, foot and mouth disease

### Sex

As for sex, recent studies suggest a higher prevalence in males than in females [4, 26, 30]. In contrast, no significant differences have been demonstrated in the genre and viral load between mild and severe cases [23].

### Etiology

The main causative agents are CVA16 and EV71 [17]. Moreover, outbreaks caused by CVA4, CVA5, CVA6, CVA9, CVA10, CVA12, CVB1, CVB3 and CVB5 have been observed [3, 9–11, 31–34]. Links have also been found between EV-4, HFMD and meningitis [22]. There is a low incidence in the cases documented in Europe, with a greater predominance of CVA16 infections. In contrast, in Asian countries such as China, the actual incidence of this disease is much higher and it has been shown that there is a large predominance of the EV71 virus in these countries, in particularly genotype C4 where the cases appear to be more severe (Table 3).

**Table 3:** Studies related to the causes of HFMD

Study	Results	Conclusions
Zhu et al. Beijing, China 2009 [27]	51 cases with HFMD 25 CVA16 positive cases 4 C4aEV71 positive cases 7 Cases neither positive for EV71 nor CVA16	In 2007 in China there was a higher incidence of HFMD caused by CVA16
Osterback et al. Turku, Finland 2009 [31]	35 cases with HFMD 34 CVA6 positive cases	CVA6 is emerging as the primary cause of the disease
Zhang et al. Pekín, Beijing 2009 [5]	70 cases with HFMD 30 Positive for enterovirus initiation: 66.7 % positive for EV71 At 4 days: 66.7 % positive for EV71 At 5 days: 12.9 % positive for EV71	Samples must be collected within 4 days after the onset of the disease, because there is more likelihood of positive viral detection
Blomgvist et al. Helsinki, Finland 2010 [3]	317 cases with HFMD 212 positive cases of CVA6 and/or CVA10	Outbreak due to new genetic variants of the Coxsackie virus, CVA10 and CVA6
Rabenau et al. Frankfurt, Germany 2010 [52]	696 cases with HFMD 88–73 % of children under 4 years are susceptible to infection 30.6 % seropositive for both viruses 43.5 % neutralizing antibodies (Ntab) 25.9 % did not have antibodies	The seroprevalence study shows a common dissemination of CVA16 and EV71 in Germany, and a comparatively higher sensitivity in the younger population
Liu et al. Nanchang, China 2011 [14]	109 cases with HFMD 90 % children under 8 years High prevalence of subgenotype C4	C4aEV71 genotype is now the more common infectious agent in China
Yang et al. Beijing, China 2011 [32]	301 cases with HFMD Enterovirus (HEV) (88.4 %), EV71 (50.4 %), CVA16 (38.3 %), CVA4 (1.1 %), CVA6 (1.1 %), CVA10 (1.1 %), CVA12 (2.6 %), CVB (5.3 %)	HFMD epidemics can persist for a long time in China, due to the different genetic variations in the composition of the virus, enteroviral characteristics of recombination and co-infection, increased travel, migration and the lack of an effective vaccine
Yan et al. Shanghai, China 2011 [30]	3208 HFMD cases EV71—86.5 %, CVA16—6.9 %, CVA16 + EV71—17.6 % Children 1–4 years 76.9 % M:F—65.3 %: 34.7 % Subgenotype C4 of EV71 circulating	The subgenotype C4 of EV71 was the main causative agent of the epidemic in Shanghai. The group most affected were children under 4 years. There was a higher prevalence in boys than in girls. High incidence of mixed infections of EV71 and CVA16
Rabenau et al. Frankfurt, Germany 2010 [52]	696 cases with HFMD 88–73 % of children under 4 years are susceptible to infection 30.6 % seropositive for both viruses 43.5 % neutralizing antibodies (Ntab) 25.9 % did not have antibodies	The seroprevalence study shows a common dissemination of CVA16 and EV71 in Germany, and a comparatively higher sensitivity in the younger population

*HFMD* hand, foot and mouth disease

### Diagnosis and differential diagnosis

Diagnosing the disease is relatively easy by looking at the clinical features of the disease.

In oral mucosa, an enanthem appears after 1–2 days and on the soft palate, inner cheeks, gums, gingival–labial groove and tongue. In our case study, the lesions appeared in less common areas such as the hard palate, tonsillar pillars and the right buccal mucosa. It consists of 5–10 small vesicles, which are very painful, covered with a yellowish pseudomembrane and surrounded by an erythematous halo. They measure between 3 and 7 mm in diameter (typically 5 mm). The enanthem breaks, forming small ulcers, as the oral epithelium is thin, which allows the vesicles to break easily during movements associated with speech and chewing. These vesicles can hinder food consumption because the tongue may be swollen and painful [13] (Fig. 1).

Skin vesicles can vary in number from a few to 100. Characteristically, they appear on the sides and back of the fingers, around the nails, around the heel and on the palm of the hand and soles of the feet. Occasionally they can appear on the knees and buttocks. Skin lesions, in our case, appeared on the sides and backs of the fingers and toes of our patient, and appeared in the locations described elsewhere. It measures approximately 3–7 mm in diameter. They are surrounded by an erythematous halo. The vesicle wall is thin and may be preceded by a maculopapular rash. They move into vesicular stage and then go on to develop scabs and ulcers [6, 13] (Figs. 2, 3).

Histopathological examination of skin vesicles revealed the presence of intraepithelial vesicles, within which there are fibrin, epithelial cells and reticular degeneration balonica, neutrophils, mononuclear cells and eosinophilic proteinaceous content [13].

In the differential diagnosis of lesions on the oral mucosa, the lesions that should be considered are primary herpetic gingivostomatitis, herpangina, erythema multiforme, aphthous stomatitis and chickenpox. It is very important to establish a correct diagnosis to avoid prescribing inappropriate drugs [17, 35].

Although it is not a serious disease, an early diagnosis is important to avoid epidemics on the pediatric population. There must be communication between the different branches of medicine and dentistry. The role of the dentist is important, as he/she is one of the professionals who must help with diagnosis when the patient seeks professional advice for painful oral lesions. Another diagnostic method studied recently by Yu et al. is the use of IgM ELISA in EV71 and CVA16 infections to correctly identify the virus causing the disease in patients [36].

### Complications

Cases of HFMD associated with onychomadesis have been documented. This relates to the fact that viral replication could damage the nail matrix and produce transient nail dystrophy. Although several cases have been documented for patients infected with CVA10, further studies are needed to determine the causative agent of HFMD associated with onychomadesis [13]. It is not necessary to treat the nails in any way except by keeping the area clean and avoiding further injury. In all of the reported cases, the nail disorders resolved themselves spontaneously over the course of several weeks [21] (Table 4).

**Table 4:** Studies related to the complications of HFMD

Study	Results	Conclusions
Ooi et al. Sarawak, Malasia 2009 [7]	725 HFMD cases Risk factors: fever ≥3 days (1), fever ≥38.5 °C (2), lethargy history (3) ≥2 factors present in 65 % of children with pleocytosis in CSF, compared to 30 % without it 2–3: S 28 %, E 89 %, PPV 79 %, NPV 50 % 1: S 75 %, E 59 %, PPV 75 %, NPV 59 %	There are three risk factors to identify children with possible neurological disorders that are easily identifiable: Fever ≥3 days duration Fever ≥38.5 °C History of lethargy
Cho et al. Seoul, Korea 2010 [23]	16 HFMD cases associated with: meningitis (10), Guillain–Barré syndrome (3), meningoencephalitis (2), poliomyelitis associated with acute flaccid paralysis (1) myoclonus (1) 11 positive cases for C4aEV71	Most neurological manifestations are caused by C4eEV71
Guimbao et al. Zaragoza, Spain 2010 [20]	27 Onychomadesis cases 24 had previously submitted HFMD Virus CVB1 and 2	Strong association between HFMD and onychomadesis. Microbiological results inconclusive
Davia et al. Valencia, Spain 2011 [19]	221 Onychomadesis cases associated with HFMD: 61 % CVA10 present in 49 % Other viruses: CVA5, 6, 16, B1 and B3, echovirus 3, 4, and 9, and EV71	The 2008 onychomadesis outbreak in Spain was associated with HFMD caused primarily by the CVA10 virus
Tian et al. China 2011 [38]	147 HFMD cases. Majority <3 years. 69.4 % males Rashes and fever ≥38.5 °C 100 % 100 % CNS involvement (lethargy 84.4 %, myoclonus 51.7 %, drowsiness 23.1 %), tachypnea 76.2 %, bradypnea 14.3 %, hypertension 15.5 %, increased chest 51.7 %, consolidation of the thorax 29.9 %, hyperglycemia 86.4 %, high levels of lactic acid 88.4 %, positive EV71 76.9 % Treatment: mechanical ventilation for 61.2 ± 12.8 h (range 40–96 h), mannitol, dexamethasone, gamma globulin, ribavirin, dopamine (58.5 %), dobutamine (51.0 %), amrinone (21.8 %) 2 % Died during hospitalization. Everyone else had a full recovery and were discharged after 14.2 ± 1.6 days (range, 12–17)	The central nervous system and cardiac system are involved in patients with severe HFMD. Fasting blood sugar and increased lactic acid levels in the majority of patients Mechanical ventilation support and drug treatment are associated with good clinical outcome of these patients
Wei et al. Taiwan, China 2011 [21]	130 HFMD cases by CVA6 Perioral rash (22 %), rash on body and/or neck (30 %), general rash (5 %), hand-foot-mouth rash (51 %), peeling of palms and soles of the feet (37 %), onychomadesis (5 %)	Patients with CVA6 associated HFMD symptoms of infection saw a broader spectrum of the destruction to the skin and deeper tissues, such as nail abnormalities and peeling
Chang et al. Taiwan. [45]	219 enterovirus 71 case subjects and 97 control children 74 % (163 of 219 cases) were complicated cases, 57 % (125 of 219 cases) are complicated cases with central nervous system involvement, and 17 % (38 of 219 cases) involved cardiopulmonary failure after central nervous system involvement Necrosis factor α promoter type II (-308 A allele), HLA-A33, and HLA-DR17 were significantly associated with enterovirus 71 susceptibility HLA-A33 was the gene most significantly susceptible to enterovirus 71. HLA-A2 was associated with the development of cardiopulmonary failure	HLA-A33, which is a common phenotype in Asian populations but is rare in white populations, was most significantly associated with enterovirus 71 infection, compared with the other candidate genes studied, whereas HLA-A2 was significantly related to cardiopulmonary failure

*HFMD* hand, foot and mouth disease, *S* sensitivity, *E* specificity, *PPV* positive predictive value, *NPV* negative predictive value

Complications have been cited on a neurological level, and early recognition of the children at risk is the key to reducing mortality and severe morbidity [7, 37, 38] (Table 3). It was therefore shown that administration of mannitol, methylprednisolone, IVIG and other supportive treatments may prevent the disease worsening in severe cases, and improve the rate of successful recovery in patients with nervous system complications [39] (Table 3).

### Severe forms

It is worth noting the large number of reports on Asian patients with HFMD and severe neurological complications caused by EV71, which can rapidly progress to fulminant cardiorespiratory failure and death. All the above studies are consistent with the fact that EV71 is the virus most responsible for serious manifestations of HFMD, and children under 2–4 years are the main group at risk of such complications. However it has not yet been determined whether neutralizing antibody responses in the early stages of infection correlate with the clinical severity of the disease in patients with EV71 [40, 41] (Table 5).

**Table 5:** Epidemiological studies on the more severe forms of HFMD

Study	Results	Conclusions
Liu et al. Shenzhen, China 2008 [37]	145 HFMD cases 124 mild cases 35 % +EV71 21 severe cases 67 % +EV71 Leukocytes and blood glucose levels of the most serious were significantly elevated. Age was less in severe cases (P < 0.05)	EV71 mainly contributes to severe HFMD High fever, elevated white blood cell count, high blood glucose concentrations and an age less than 4 years should be used to predict severe cases
Ang et al. Singapore 2009 [49]	Annual incidence rate of HFMD rose from 125.5 in 2001 to 435.9 in 2007 per 100,000 habitants	HFMD remains a major public health problem in Singapore. Must maintain a high degree of vigilance, particularly for EV 71
Sarma et al. W. Bengal, India 2009 [51]	38 HFMD cases. Children 1–12 years M:F—21:17. Oral lesions 86.6 %. High incidence of EV71	An alarmingly high prevalence of EV71. No significant differences between boys and girls
Wu et al. Hangzhou, China 2010 [24]	28 HFMD cases Severe cases <2 years 88.89 % Severe patients with EV71—92.86 % Mild patients with EV71—36.51 %	Children under 2 with EV71 are the highest risk group for developing the severe form of the disease
Suzuki et al. Tokyo, Japan 2010 [53]	199 HFMD cases. Severe 96. Mild 103 No differences in sex, age, family history. No significant association between the center of care and disease presentation	There is no clear association between center of care and presentation. More studies are needed in this regard
Zeng et al. Shangai, China 2012 [26]	28,058 HFMD cases Neurological disorders, pulmonary edema and hemorrhage 2.6 % 0.04 % deaths More cases in summer and higher frequency in boys than girls 1–4 years 82.27 % EV71 positive: 99.17 % severe cases in 2009, 86.31 % in 2010, 100 % of patients with neurological affectation, edema, pneumonia and hemorrhage	Dominant EV71 circulation led to the outbreak of HFMD and the occurrence of severe and fatal cases in China EV71 is associated predominantly with severe cases of the disease
Wang et al. Beijing, China 2011 [2]	Children 1–3 years OR >2.3 Serious illness OR >1.4 Death OR >2.4 Children OR = 1.56 boys compared to girls 1 day delay in diagnosis associated with severe disease increase (40 %) and the probability of death (54 %) EV71 association with severe disease OR = 16 and death OR = 40, regarding CVA16	HFMD is transmissible especially among preschoolers. Enterovirus 71 was responsible for the most serious cases and deaths in China. The mixture of asymptomatic infected children in school might have contributed to the spread of infection. Diagnosis is very important to reduce the high mortality rate
Fang et al. Shaoxing, China 2014 [43]	19 studies meta-analysis Duration of fever ≥3 days [odds ratio (OR) 10.09, 95 % confidence interval (CI) 6.22–16.35], body temperature ≥37.5 °C (OR 4.91, 95 % CI 1.26–19.18), lethargy (OR 7.75, 95 % CI 3.78–15.89), hyperglycemia (OR 2.77, 95 % CI 2.06–3.71), vomiting (OR 8.83, 95 % CI 1.05–74.57), increased neutrophil count [weighted mean difference (WMD) 0.61, 95 % CI 0.52–0.70], enterovirus 71 (EV71) infection (OR 5.13, 95 % CI 3.11–8.46), young age (WMD—0.44, 95 % CI 0.69–0.19), and home care (OR 1.65, 95 % CI 1.26–2.17) were significantly related to the risk of severe HFMD A confirmed diagnosis at first visit to hospital significantly decreased the risk of severe HFMD (OR 0.30, 95 % CI 0.09–0.99) not find an association between oral rash (OR 1.07, 95 % CI 0.82–1.39), increased leukocyte count (WMD 0.51, 95 % CI 0.05–1.06), male sex (OR 1.06, 95 % CI 0.91–1.24), or living in a rural area (OR 1.39, 95 % CI 0.95–2.02) and the risk of severe HFMD	Duration of fever ≥3 days, body temperature ≥37.5 °C, lethargy, hyperglycemia, vomiting, increased neutrophil count, EV71 infection, and young age are risk factors for severe HFMD. A confirmed diagnosis at first visit to hospital can significantly decrease the risk of severe HFMD
Chen et al. Shangai, China [25]	The mean serum VA concentration for all patients was 0.73 ± 0.26 mmol/L, and 237 (52.7 %) of them presented low concentrations (≥0.7 mmol/L). Both serum concentrations of VA and IFN-a in the patients with complications were significantly lower than in patients without complications (P < 0.01) The decreased concentrations of IFN-a and EV71-IgM were positively related to lower VA levels (correlation coefficient ¼ 0.58 and 0.41, respectively, P < 0.001)	VA status is associated with the antiviral immunity and pathogenetic condition of HFMD in young children. The children with HFMD mostly presented low VA concentrations and simultaneously had lower serum IFNa levels, decreased immune antibody production and more severe illness

*HFMD* hand, foot and mouth disease

In a meta-analysis by Li et al. [42], in mainland China and Taiwan, concluded that blood counts increased glucose and leukocytes in severe forms of the disease, are a tool that can help physicians to anticipate the diagnosis and treatment of these patients efficiently.

Fang et al. in 2014 [43], in a meta-analysis of Risk factors of severe HFMD, concluded that Duration of fever ≥3 days, body temperature ≥37.5 °C, lethargy, hyperglycemia, vomiting, increased neutrophil count, EV71 infection, and young age are risk factors for severe HFMD. A confirmed diagnosis at first visit to hospital can significantly decrease the risk of severe HFMD.

Recently, cellular receptors have been identified, along with host factors that stimulate EV71. Several lines of research suggest that the scavenger receptor class B, member 2 (SCARB2) plays a crucial role in EV71 infection and later development into severe forms of the disease [44].

### Vaccine

The EV71 vaccine is also important for preventing infection. Recent studies show that both serotypes of human leukocyte antigen, HLA-A33 (class I) and HLA-DR17 (class II), have significant associations with EV71 infection, suggesting that cellular immune response may play an important role in human immunity against EV71 infection [45]. It was also found that the most severe cases of EV71, characterized by pulmonary edema, had lower cell specific antigen cytokines (Th1) and a lower lymphocyte proliferation response to the EV71 antigen in comparison with milder cases. This suggests that cellular immune response correlates with the clinical severity of HFMD. Namely, induction of cellular and humoral immunity should be considered when developing vaccines and further analyzed in clinical trials [44]. In 2010, effective antiviral agents and vaccines against this virus was under development [46], Another study, in 2012 presented the first construction and characterization of an infectious cDNA clone of CVA16. The availability of this infectious clone also improved virological investigations and CVA16 vaccine development [42]. For 2015, formalin-inactivated EV71 (FI-EV71) vaccines have been developed evaluated in human clinical trials, and were found to elicit full protection against EV71, and bivalent FI-EV71/FI-CVA16 vaccines have been found to elicit strong neutralizing antibody responses against both viruses in animal models, but nevertheless, there is currently no approved vaccine or antiviral substance available for the prevention or treatment of EV71 infection [47, 48].

### Prevention

On another note, the HFMD patient is potentially contagious for the duration of the initial symptoms, until the vesiculobullous skin lesions disappear. Therefore, patients diagnosed with HFMD should be excluded from group participation until the fever and skin and mucosal lesions have disappeared. Furthermore, the virus is known to be shed in the feces for several weeks. It is therefore necessary for patients and family members to know that the disease is spread through direct contact with blisters and secretions from the nose and throat, as well as through hands or utensils contaminated with the feces or secretions of infected people. It is therefore important to emphasize washing hands well and to avoid sharing contaminated utensils. In the case we presented, up to four adults who were in direct contact with the infected child suffered from the disease, as in the case described by Shin et al. [6]. To prevent the infection from spreading to children and vulnerable adults, doctors and dentists should be aware that HFMD can also occur in immunocompetent adults [6]. It has also been shown that public health measures such as closing schools and hygiene campaigns are effective in reducing the incidence rate of HFMD [49].

Due to multiple pathogenic compositions, enteroviral recombination, co-infection, spreading and the lack of an effective vaccine, outbreaks of the infection can persist for a long time. It is therefore vitally important to carry out epidemic surveillance of this disease and its complications, and consequently clinicians should inform health authorities to promote citizen participation in dealing with large epidemics of HFMD [50, 51].

Our case was resolved spontaneously with symptomatic treatment without complications over a period of 7–10 days, as reflected in other articles [6, 13].

## Conclusions

HFMD is a typical childhood illness, but it also occurs in adults, which should be kept in mind due to the possibility of outbreaks, with emphasis on medical history and the overall clinical picture, to avoid inadequate treatments with antibiotics.

Dentists have an advantage over other professionals in diagnosing the disease, as lesions in the oral cavity are the main symptoms that affect the patient, and the dentist is therefore frequently one of the first professionals to be consulted. Thus, knowledge of the disease and early detection prevents the infection from spreading to other children and adults. Similarly, dentists have a key role in educating patients by recommending good oral hygiene to minimize the spread of the disease.

A surveillance system to predict future outbreaks, appropriate public health measures and research into vaccine development are of vital importance to control HFMD.

## Consent

Written informed consent was obtained from the patient for publication of this case report and any accompanying images.

## Abbreviations

HFMD: hand foot and mouth disease
EV: enterovirus
CV: coxsackievirus
IVIG: intravenous immunoglobulin
SCARB2: scavenger receptor class B, member 2
FI-EV71: formalin inactivated enterovirus 71
FI-CVA16: formalin inactivated coxsackievirus 16
